# Cutaneous Leishmaniasis Caused by *Leishmania killicki,* Algeria

**DOI:** 10.3201/eid2003.13-1152

**Published:** 2014-03

**Authors:** Arezki Izri, Amina Bendjaballah, Valérie Andriantsoanirina, Rémy Durand

**Affiliations:** Hôpital Avicenne–Assistance Publique-Hôpitaux de Paris, Bobigny, France (A. Izri, V. Andriantsoanirina, R. Durand);; Hôpital de Hadjout, Hadjout, Algeria (A. Bendjaballah)

**Keywords:** cutaneous leishmaniasis, Leishmania tropica, Leishmania killicki, Leishmania infantum, Algeria, parasites

**To the Editor:** Cutaneous leishmaniasis (CL) is a widespread and resurging vector-borne disease caused by a protozoan parasite belonging to genus *Leishmania* (*1*). After Afghanistan, Algeria is the second largest focus of CL in the world. Although CL is a serious public health problem in Algeria, few data are available from this country. 

During 2004–2008, an average of ≈44,050 CL cases were reported per year, and the estimated annual incidence ranged from 123,300 to 202,600 cases. Two main forms of CL have been described for more than a century in Algeria, the zoonotic, caused by *L. major* and the sporadic, caused by *L. infantum*. Since 2004, 11 strains belonging to the *L. tropica* complex, including *L. killicki* (*2*), were identified in 1 focus in the northern part of the Sahara (*3*) and in 2 foci in the northeastern Algeria (*4*,*5*). We report here a recent outbreak of CL, including infection with *L. killicki* strains, in the Tipaza area of northern Algeria.

Patients who sought treatment at Hajout hospital in Hajout, Algeria (a community of ≈51,000 persons), from January 2010 through April 2013 with cutaneous lesions consistent with leishmaniasis, underwent clinical examination. For each patient (146 total), we collected epidemiologic data (geographic origin, traveling history, especially to other leishmaniasis-endemic areas) and clinical data (number and size of lesions and clinical forms). Informed consent was obtained from all patients or their legal guardians. A particular characteristic of the infections was the unusual duration of some episodes, one of which persisted for >4 years, which is compatible with leishmania recidivans (*6*). 

Microbiological data were obtained as follows. Tissue samples, obtained by scraping the internal border of skin lesions from patients, were smeared onto a glass slide, fixed with methanol, stained with Giemsa, and examined by microscopy. Slides showing *Leishmania* amastigote forms were then processed further for molecular analyses. The immersion oil used to examine each slide was wiped off the smear with tissue paper, and then the dry smear was scraped from its slide by using a sterile scalpel. DNA extraction from smear scrapings was performed with the NucleoSpin Tissue kit (Macherey-Nagel, Düren, Germany). Species identification was performed by amplifying the topoisomerase II gene, followed by DNA sequencing (*7*).

In total, 60 patients exhibited *Leishmania-*positive cutaneous lesions as determined by microscopy. The topoisomerase II gene was successfully amplified and sequenced from samples from 38 patients. *Leishmania* species were identified by comparing sequences with those of the reference strains *L. infantum* MHOM/FR/78/LEM75, *L. killicki* MHOM/TN/80/LEM163, and *L. major* MHOM/MA/81/LEM265 (*7*). *L. infantum* was identified in 36 cases and *L. killicki* in 2 cases (Figure). No *L. major* isolates were found in this series.

**Figure:**
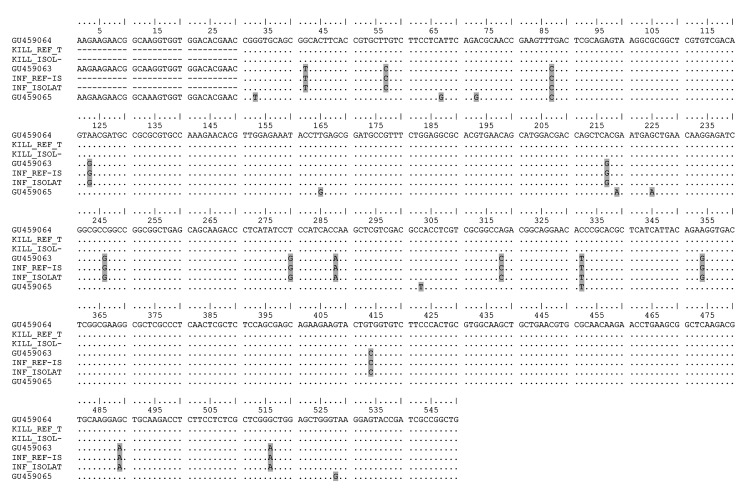
Alignment of topoisomerase II nucleotide sequences of *Leishmania killicki*, *L. infantum*, and *L. major*. Point mutations discriminating *Leishmania* species are outlined on a gray background. The references strains are GU459063: *L. infantum* MHOM/FR/78/LEM75; GU459064: *L. killicki* MHOM/TN/80/LEM163; GU459065: *L. major* MHOM/MA/81/LEM265 ; KILL_REF_T: *L. killicki* and INF_REF-IS: *L. infantum*, strains genotyped by the *Leishmania* National Reference Center, Montpellier, France. The isolates are: KILL_ISOL-: *L. killicki* (n = 2); INF_ISOLAT: *L. infantum* (n = 36).

The low proportion of *L. killicki* strains was similar to that found recently in the Annaba focus in northeastern Algeria (*5*). However, the observation of a new focus of CL and *L. killicki* as etiologic agent may indicate a modification of the epidemiology of CL in Algeria. This focus, located far from other previously described areas where the *L. tropica* complex is endemic, may reflect geographic spread of this complex in Algeria. 

The results of this study can be placed in a larger framework as well. Since 2004, strains in the *L. tropica* complex have been increasingly reported as responsible for CL in Mediterranean countries, in the Near East and Middle-East (*2*), possibly in relation to changes in environmental conditions. Urbanization and/or climatic changes that have occurred in recent years could have played a role in the spread of the disease. The cases reported here were observed in urban areas, which suggests transmission according to an anthroponotic mode.

Each species responsible for CL has its own epidemiologic pattern. Clinicians must be aware of the specificity of leishmaniases that may be encountered in North African countries. *L. tropica* complex lesions heal spontaneously over a period of 12 months or more, a duration longer than for *L. major* infections (*8*). *L. tropica* infections are also less responsive to treatment compared to infections with other Old World *Leishmania* species. In addition, *L. tropica* may cause leishmania recidivans. This type of CL, appearing often years after the initial infection showed signs of complete resolution, manifests as papules that transform slowly into a spreading granuloma resembling lupus vulgaris (*6*). *L. tropica* can also produce visceral infections on rare occasions, resulting in unexplained systemic illness, including classic symptoms of visceral leishmaniasis, in persons returning from areas where this *Leishmania* complex is endemic (*9*).

Other epidemiologic studies are required to detect additional foci, including those of the *L. tropica* complex, that may coexist with those of *L. infantum* and *L. major* in Algeria. Travelers to North Africa should also be informed about the existence of this spreading disease (*10*).
